# Supplementary material for: Heterologous Biosynthesis of Health-Promoting Baicalein in Lycopersicon esculentum
Source: Molecules. 2022 May 11;27(10):3086. doi: 10.3390/molecules27103086 (PMC9146059; doi:10.3390/molecules27103086)
Supplement: Supplementary file 1 [file molecules-27-03086-s001.zip › molecules-1717509-supplementary.pdf]

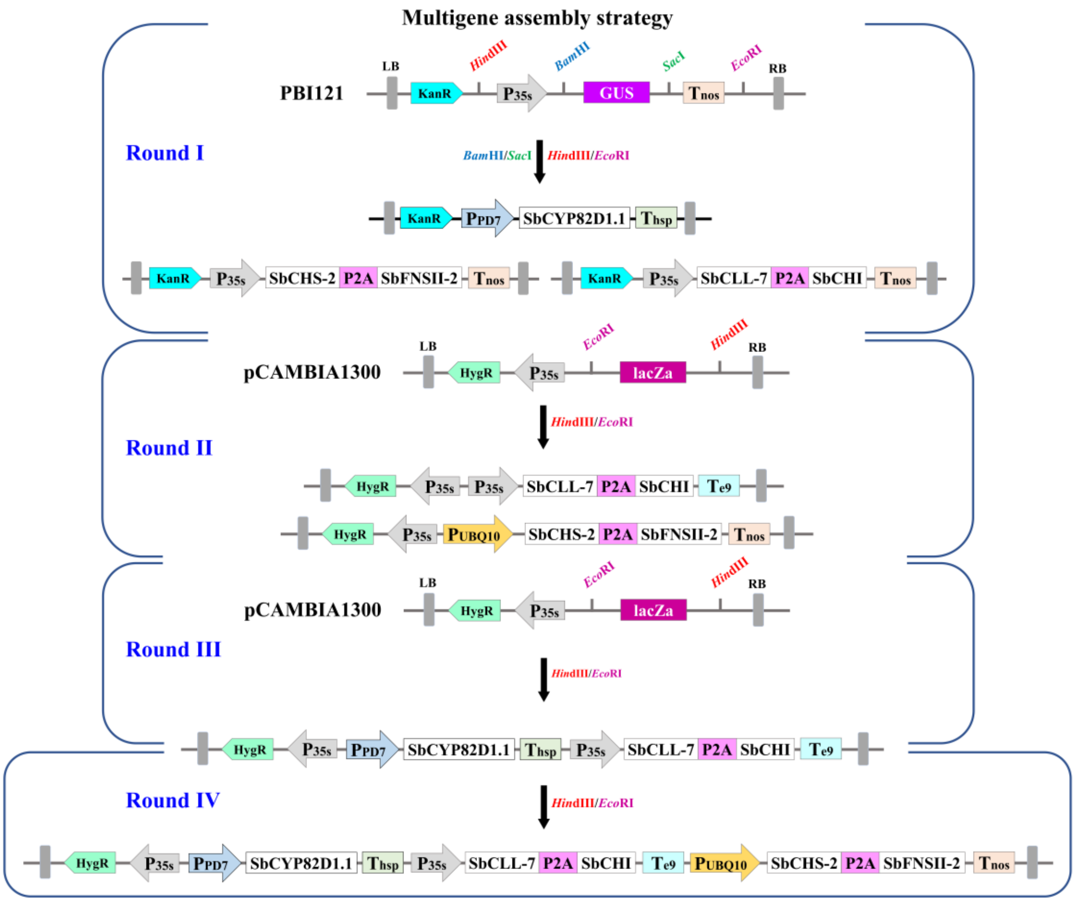

Figure S1. The multigene assembly strategy for baicalein biosynthesis genes. Total 4 rounds of multigene assembly have been performed in this experiment. The amino acid sequence of P2A is GSGATNFSLLKQAGDVEENPGP. *Bam*HI/*Sac*I and *Hind*III/*Eco*RI are restriction enzyme sites. HygR was hygromycin, KanR was kanamycin.

ATAGGCAACCGTGGACTTCTTCACTAGCTCATCGAGATAGCAATGCCACTAGCTAATT
TCTTACGTTGTATCTTATTTGTTTTACTTTGGGGCATGACATGGTTAAACCCCATCAAA
GAGAAAGTGTTTCATCCACTAGTCAATATACGAATTCATTCGAATTTATCCCTGTAAA
TCCTAGTTCTTAGGATGAACTGGTGTAATAAACAGCAAAAAAAAAAAAAAAAAAAAA
AAATCTGGAATCATTCGACCACCTCAATAAACTAAAGCTACCAATTACCACAATATA
GTCTTCCATATCCACTTAGATATAAAAGATAAAAGTAAACAAATATTAAATTTCATAT
GCACGCATAGGAAACTCATGATCTTATCTTTTAAATAGACATCTAGTTTTCTTAGGTT
ATAAATAGACATTTTGTCCTAGAACTTCTTCACTACTAAAACCTAGCCTCAAATGGAG
TTGAGCTCTGTCATCTATGGCGCCATCGCCTTGCTCTCTCTTTTCTACTGTTACCTACA
CTTCTCCAAGCCAAAAAAGAGTTCCCTGAACGCGCCGCCGGAGGCTGGCGGCGCGCG
CTTCATCACCGGCCACCTCCACCTGATGGACGGCCGCTCCGCCTCCGACAAACTTCCT
CACATAAACCTGGGTTTGCTTGCAGACCAGCACGGCCCAATCTTCACGATCAGGCTG
GGCGTGCACCGAGCCGTGGTGGTGAGCAGCTGGGAGCTAGCCAAGGAGATCTTCACC
ACCCACGACACGGCGGTCATGGCTCGGCCCCGGCTCATAGCCGACGACTACCTGAGC
TACGACGGAGCCTCGCTTGGGTTCTCACCCTACGGACCATACTGGCGCGAAATCCGC
AAGCTGGTCACCACCGAGCTGCTCTCGGCTCGCCGGATCGAGCTGCAGCGAGCCACG
CGTGTGCGTGAGATCACGCAGTTCACCGGCGAGCTCTACAAGCTGTGGGAGGAGAAG
AAAGACGGGTCTGGGAGGGTTTTGGTGGACATGAAGCAGTGGCTGGGGAACTTGAGC
CTCAATCTGGTGTCGAGGATGGTGGTGGGCAAGAGGTTCTACGGCGGCGACGACTCG
GAGACGACCAAGAGGTGGCGGGGCGTCATGAGGGAGTTCTTCCAGCTCATCGGACAG
TTCATCCCCGGCGACGGGCTTCCGTTTCTCCGGTGGCTCGACTTGGGTGGGTTCGAGA
AGAGGACGAGGGACACTGCCTATGAGCTGGATAAGATCATCGCAATGTGGCTGGCGG
AGTATCGGAAAAGGGAATATTCCGGCGACGACAAGGAGCAGTGCTTCATGGCCCTCA
TGCTCTCACTCGTGCAAGCTAATCCCACTCTGCAACTCCACTACGATGCTGATACCAT
CATCAAAGCTACTTGCCAGGTTTTGATATCGGCGGCAAGTGACACGACGACGGTGAT
CCTAATCTGGGTAATTTCCCTCCTACTAAACAATGCTGATGTCCTAAAAAAGGTTCAA
GAAGAACTGGACGAACAAGTGGGGAGAGAAAGACGAGTGGAAGAATCGGACATAA
GCAATTTACCCTACCTCCAAGCGGTGGTGAAGGAGACAATGAGGCTATACCCTCCGG
CCCCCTTCGCCGGAGTACGAGCCTTCAGCGAAGACTGCACCGTGGGAGGCTACCACA
TCCAGAAAGGCACGTTTTTGATAGTCAATCTGTGGAAGCTGCATCGAGACCCTCGTGT
GTGGTCCGATGATGCCTTGGAGTTCAAACCACAACGGTTTTTTGACAAAAAGGTGGA
GGTTAAGGGTCAGGACTTCGAGTTGATGCCATTTGGTGGTGGTCGAAGAATGTGTCCC
GGCTCGAACCTGGGCATGCACATGGTGCACTTTGTGCTGGCTAACATATTGCAGGCCT
TTGACATAACCACTGGGTCCACTGTGGATATGACCGAGAGTGTTGGGTTGACCAACA
TGAAAGCCACACCACTTGATGCTATTCTCACTCCAAGGTTGTCACCTACTCTATATTA
AATATGAAGATGAAGATGAAATATTTGGTGTGTCAAATAAAAAGGTTGTGTGCTTAAGT
TTGTGTTTTTTTCTTGGCTTGTTGTGTTATGAATTTGTGGCTTTTTCTAATATTAAATGAA
TGTAACATCTCATTATAATGAATAAACAAATGTTTCTATAATCCATTGTGAATGTTTTGTT
GGATCTCTTCTCCAGCATATAACTACTGTATGTGCTATGGTATGGACTATGGAATATGATT
AAAGATAAGTGAGACTTTTCAACAAAGGGTAATATCCGGAAACCTCCTCGGATTCCATT
GCCCAGCTATCTGTCACTTTATTGTGAAGATAGTGGAAAAGGAAGGTGGCTCCTACAA
ATGCCATCATTGCGATAAAGGAAAGGCCATCGTTGAAGATGCCTCTGCCGACAGTGGT
CCCAAAGATGGACCCCCACCCACGAGGAGCATCGTGGAAAAAGAAGACGTTCCAACC
ACGTCTTCAAAGCAAGTGGATTGATGTGATATCTCCACTGACGTAAGGGATGACGCAC

AATCCCACTATCCTTCGCAAGACCCTTCCTCTATATAAGGAAGTTCATTTCATTTGGAGA
GAACAATGGAGAAATCGGGCTATGGCCGTGATGGAATATACAGATCATTGAGGCCACC
GTTGTATCTTCCCAAAAATCCCAATCTTTCACTTGTTTCGTTTCTCTTCAGAAACGTTTC
GTCTTTTGCTGATAACCGAGCTCTTGTGGATGCACATACTGGGGAAACCTTAACCTTCG
CGCAGTTTAAATCCACGGTTTCAAAAGTCGCCCATGGTTTACTTCAACTGGGCATCAAG
AAAAACGACGTCGTGCTCATCTTCGCTCCCAATTCGATCGAGTTCCCAATATCTTTCTTT
GGGGTTATCGCCACTGGAGCCGTCGCCACCACGGTCAACCCCATGTATACTGTCTCGG
AGCTATCCAAACAGGTTCAAGATTCTACTGCTAAGCTTATAATTACTGTTGAAGGTCTG
TTGCCTAAAGTTAAGGATTTGGTTTGCCTGTTATTCTTCTGGGAAATGAAAAGAAATC
AGTTTTGCCCATTGGGAGAATCCCTTCAATCACTTCATTTTCTGAGCTTGTGAACAATG
AAGGGTTGGATTTAGATAGTAATGTTTATGGAATCAAGCAAGATGATACAGCTGCTTTG
TTGTACTCATCCGGCACCACGGGTATGAGCAAGGGCGTTATACTTACTCATAGGAACTT
CATAGCTTCCTCGTTGATGATCACTGCTGATCAAGAATCGGCTGGTGAAATGCACAATG
TGTTTCTCTGTGTTCTGCCTATGTTTCATGTTTTTGGTCTTGCAGTGATCATGTTATCACA
GCTGCAACGTGGAAACACCATTGTTTCGATGTCGAAGTTTGATTTTGAAATGATTTTGA
AGACGGTGGAGAAGTATGGGATCACGCATATGTGGGTTGTGCCGCCAATTATATTAGCT
TTGGCCAAAAACAATGTGGTCAAGAAGTATAATTTGTCATCCTTGCAGCAGATTGGATC
GGGGGCGGCTCCACTTGGGAGAGAATTGATGCAGGAATGTGCAAAGAATTTCCCTCAA
GCTGTTGTCGTTCAAGGTTATGGAATGACAGAAACCTGTGGAATTGTCTCCGTCGAGA
ATGTGTATGCAGGGCCACGACATTCTGGTTCTGCTGGTACACTTGTTCCTGGTGTGGAG
TGTCAAATAGTTAGTGTTGATAAATTGAAGCCTCTTCCTCCTGGACAGTTGGGTGAAAT
ATGCGTTCGAGGACCCAATATGATGCAAGGTTATTTTAAGAATCCACAGGCTACGAAGC
TTACAATAGATAAACAGGGTTGGGTCCATACTGGAGATCTTGGATACTTCGATGATGAG
GGTCAGCTCTTTGTTGTTGACCGTATTAAAGAGCTCATTAAGTACAAAGGTTTTCAGGT
TGCTCCAGCGGAGCTTGAAGGGCTACTCGTCTCTCATCCCGAGATATCAGATGCTGTTG
TCATCCCGTTTCCTGATGCTGAAGCTGGTGAGGTCCCGATTGCTTATGTTGTCCGCGCT
CCTAATAGCTCACTGTCTGAAGAAGATATCAAGAACTTCATTGCTGAGCAGGTGGCTCC
TTTTAAAAGATTGAGAAGAGTTACATTCATAAACAGTGTCCCGAAATCCGCAGCTGGG
AAAATACTTAGAAGAGAACTCATCGAAAAGGTCCGATCGAAGTTAGGAAGCGGAGCT
ACTAACTTCAGCCTGCTGAAGCAGGCTGGAGACGTGGAGGAGAACCCTGGACCTATG
TCTGCTTCGCCATCCGTCACCAAAGTCCTAGTCGAATCCATCGAGTTCTCGCCGGCCGC
AAAGCCGCCGGGATCCTCCAACACCTTATTCCTCGGCGGCGCCGGGGTGAGAGGAATG
GAGATCCAGGGGAACTTCGTGAAGTTCACGGCGATCGGTGTTTACTTGGAAGACAGCG
CCGTTCCGTCTCTCGCCGTTAACTGGAAGGGCAAGACCGCCGAGGAGTTGACGGAGT
CAGACGACTTTTTCAGGGAAATCGTGTCTGGTCCTTTCGAGAAATTCACCAAGGTGAC
GATGATTCTGCCATTGACGGGGAAGCAATACTCGGAGAAAGTTGCAGAGAACTGCGTT
GCGTACTGGAAAGCCGTAGGGAAATACACGGATGCTGAATCGGAAGCAATCGACAAG
TTTCTCCAAGTGTTCAAGGACGAAACGTTCGCGCCTGGAGCTTCCATCCTCTTCACCC
AATCGCCGGCCGGCTCCTTGACGATTAGCTTCTCGAAAGACGGATCGATTCCAGAGCA
AGGGAAGGCAGTAATAGAGAACAAACAGCTGTCGGAGGCAGTGCTGGAGTCGATCAT
CGGAAAGCATGGTGTGTCGCCGTCGGCGAAGCAGAGTTTGGCGGCAAGACTATCGGA
GTTGTTTTAAAGCTTTCGTTCGTATCATCGGTTTCGACAACGTTCGTCAAGTTCAATGC
ATCAGTTTCATTGCGCACACACCAGAATCCTACTGAGTTTGAGTATTATGGCATTGGGA
AAACTGTTTTTCTTGTACCATTTGTTGTGCTTGTAATTTACTGTGTTTTTTATTCGGTTTT

CGCTATCGAACTGTGAAATGGAAATGGATGGAGAAGAGTTAATGAATGATATGGTCCTT
TTGTTCATTCTCAAATTAATATTATTTGTTTTTCTCTTATTTGTTGTGTGTTGAATTTGAA
ATTATAAGAGATATGCAAACATTTTGTTTTGAGTAAAAATGTGTCAAATCGTGGCCTCTA
ATGACCGAAGTTAATATGAGGAGTAAAACACTTGTAGTTGTACCATTATGCTTATTCACT
AGGCAACAAATATATTTTCAGACCTAGAAAAGCTGCAAATGTTACTGAATACAAGTATG
TCCTCTTGTGTTTTAGACATTTATGAACTTTCCTTTATGTAATTTTCCAGAATCCTTGTCA
GATTCTAATCATTGCTTTATAATTATAGTTATACTCATGGATTTGTAGTTGAGTATGAAAA
TATTTTTTAATGCATTTTATGACTTGCCAATTGATTGACAACATGCATCAAGTCGACGAG
TCAGTAATAAACGGCGTCAAAGTGGTTGCAGCCGGCACACACGAGTCGTGTTTATCA
ACTCAAAGCACAAATACTTTTCCTCAACCTAAAAATAAGGCAATTAGCCAAAAACAA
CTTTGCGTGTAAACAACGCTCAATACACGTGTCATTTTATTATTAGCTATTGCTTCACC
GCCTTAGCTTTCTCGTGACCTAGTCGTCCTCGTCTTTTCTTCTTCTTCTATAAAACA
ATACCCAAAGAGCTCTTCTTCTTCACAATTCAGATTTCAATTTCTCAAAATCTTAAAA
ACTTTCTCTCAATTCTCTCTACCGTGATCAAGGTAAATTTCTGTGTTCCTTATTCTCTC
AAAATCTTCGATTTTGTTTTCGTTCGATCCCAATTTCGTATATGTTCTTTGGTTTAGATT
CTGTTAATCTTAGATCGAAGACGATTTTCTGGGTTTGATCGTTAGATATCATCTTAATT
CTCGATTAGGGTTTCATAGATATCATCCGATTTGTTCAAATAATTTGAGTTTTGTCGAA
TAATTACTCTTCGATTTGTGATTTCTATCTAGATCTGGTGTTAGTTTCTAGTTTGTGCG
ATCGAATTTGTCGATTAATCTGAGTTTTTCTGATTAACAGATGGTGACAGTTGAAGAA
TTCCACCGGGCAACACGGGCAGAGGGCCCCGCCACCGTCTTGGCCATCGGCACGGCC
AACCCCCCAAACTGCGTTGAGCAGTCTACTTATGCTGATTACTACTTTCGCATTTGTAA
AAGTGAACACTTGACTGATCTTAAAAAGAAGTTTAGCCGCATGTGCGAAAAGTCTGGC
ATCAAGAAACGTTACATGCATTTAACGGAGGAGTTTCTGAAGGAGAACGACAACTTCA
CGGCGTACGAGGCTCCGTCACTGGATGCCCGGCAGGACATAGTGGTGGTGGAGATCCC
GAAGCTGGGGAAGGAGGCTGCCCAGAAGGCGATCAAGGAATGGGGGCAGCCCAAGT
CCAAGATCACCCACGTCATCTTCTGCACTACCAGCGGCGTCGACATGCCCGGCGCAGA
CTACCAGATCACCAAGCTCCTCGGCCTCCGCCCCTCCGTCAAGCGCTTCATGATGTACC
AGCAGGGCTGCTTCGCCGGCGGCACGGTGCTCCGCATGGCCAAGGACCTGGCTGAGA
ACAACGCCGGAGCTAGGGTTCTTGTCGTCTGCTCCGAGATCACCGCCATCACTTTCCG
GGGGCCCAGCGACACCCACCTCGACAGCCTGGTCGGCCAGGCCCTGTTCGGCGACGG
CGCCGGGGCGGTCATCGTTGGATCCGACCCCATCGTGGGGGTGGAGCGGCCGCTCTTC
CAGCTGGTCTCGGCGGCGCAGACGATTCTCCCTGACAGTGAGGGCGCCATTGACGGCC
ACGTCCGCGAGGTCGGGCTGACCTTCCACCTCCTCAAGGACGTCCCCGGGCTGATCTC
CAAGAACATCGAGAAGAGCCTCAAGGAGGCCTTCGCGCCGCTGGGCATCTCCGACTG
GAACTCCCTCTTCTGGATCGTGCACCCCGGAGGTCCCGCCATTCTCGACCAGGTGGAG
GAGAAGCTCGGGCTCAAGCCCGAGATCATGGTCCCAACCAGGCACGTGCTTAGCGAAT
ACGGGAACATGTCCAGCGCCTGCGTTCTCTTCGTGATGGATGAGATGAGGAAGGCCTC
CGCCAAGGACGGCTGCACCACCACCGGAGAAGGGAAGGACTGGGGGGTTCTTTTCGG
CTTCGGCCCGGGCCTCACCGTTGAGACTGTAGTTCTGCATAGTGTGCCTCTCAATGGTT
CAGGTGCCACTAATTTTTCTCTCTTGAAACAGGCCGGTGACGTTGAAGAGAACCCAGG
TCCAATGGAAGTCACACTGAATGTGGCCCTCCTCCTCTCCGCCGCCGTCTGCCTGA
TGGTCTTCACCGGTAAGAGGCGGCGGCGCCTCCCCAACCCTCCGGGCCCGTTCCCCCT
CCCACTCATCGGGAACCTGAACCTGGTGAGCCCGAGGCTGCACCACACCTTCCACATG
CTGGCTCAGCGGTACGGGCCTATCATGAAGTTCCGGCTGGGGTCCATCCCCTGCCTGGT

GGTCTCCACGCCGGAGCTGGCCAAGGACATCCTCAAGACCCACGAGCTCATTTTCTCG
TCGCGGGTCAAGTCCACCGCCATCGACATCGTCACCTATGGCGTCTCCTTCGCCTTCTC
CCCCTACGGGCCCTACTGGAAGTACATCAAGAAGCTGTGCACCTACGAGCTGCTGGGC
TCGAGAATGCTGAACCACTTCGAGCCACTCAGGGCCCTGGAGGTGCGCGAGTTTCTCA
AGGATGTGATGGCTATGGGGAAGGCTGGGAAGAGCTTCAACGTCACTGAGGAGCTGA
TGAAGCTCACCAGCAATGTCATGTCCAACATGATGCTCAGCATCAGAGCCGCCGAGTC
GGAGGAGCAGGCCGAGGTGGCCAGGACTCTCATTCGAGAAGTTTCCCAGCTCTTCGG
GGAGTTTGATTTCGGAGATATGCTCTGGTTTTGCAAGAGCTTCGACTTTCAGGGGATCA
AGAAGAGGTCCAAGGACATCAAGGTCAGGTACGATGCTTTGCTTGAAAAGATTCTCAC
AGACAGAGAGAACGTCCGACGGCAAAACGGCGTCGTGGAGCCCAAGGACATGCTTG
ACATGTTTCTTGATATCATGGAGGGCGGCAAAACCGACGTGGAATTCACCAGGGAACA
TCTCAAAGCTGTCATTTTGGACTTCTTGACCGCGGGTACGGACACAACCGCCATAACG
GTGGAATGGGTTCTTGCGGAGCTGATGAACAGCCCTAAAGCGATGAAGAAAGCTCAA
GATGAAATGGACAGAGTGGTTGGAAGAGAGAGAATGATGGCAGAATCGGACGCCCCA
AATCTGCCCTACTTCCTGGCCATCATAAAGGAGACCTTCAGGCTCCACCCTCCCATCCC
CTTGATCATCCGCCGATCCATCGAAGACTGCGTCATCGACGGCTACCACATTCCGGCGG
ACACCCTGGCCTTCATCAACGTGTGGTCGATGGGGCGGAACGAAAAGTATTGGGACAG
CCCCCTGTCGTTCAGACCCGAGAGATTCTTGGAGGGGGACAATGCCGCCATTGATATC
AAAGGGATGCACTTCGAGCTGCTGCCCTTCGGCTCTGGCAGGAGGGGCTGCCCGGGG
ATGTTGTCCGCCATCCAGGAGGTGCTCATTATAGCCGGGACCGTCATCCAGTGCTTCGA
CTGGGAGCAGGCTGACGGGTCGGGTCGGGTCGACATGAGCGAGCGCCCCGGCCTGAC
CACGCCCCGGGAAATCGATCTGGTTTGCCGGGTCGTGCCCCGGGTCGACGAGAGGGTT
ATTTCCGGGCACTGAGATCGTTCAAACATTTGGCAATAAAGTTTCTTAAGATTGAATCC
TGTTGCCGGTCTTGCGATGATTATCATATAATTTCTGTTGAATTACGTTAAGCATGTAATA
ATTAACATGTAATGCATGACGTTATTTATGAGATGGGTTTTTATGATTAGAGTCCCGCAAT
TATACATTTAATACGCGATAGAAAACAAAATATAGCGCGCAAACTAGGATAAATTATCGC
GCGCGGTGTCATCTATGTTACTAGATC

Figure S2. The nucleotide sequence of insertion fragment in the multigene vector FCC-CF. The baicalein biosynthesis genes *SbCYP82D1.1* (blue), *SbCLL-7* (pink), *SbCHS-2* (black), *SbCHI* (purple) and *SbFNSII-2* (gray) were showed in this graph. P2A peptides sequences was highlighted in red. The nucleotide sequences of two 2A peptides was different, but there was the same amino acid sequence of GSGATNFSLLKQAGDVEENPGP. *SbCYP82D1.1* gene was controlled by *AtPD7* promoter (red), and the terminator was heat-shock protein 18.2 terminator (Thsp) (highlighted in light yellow). *SbCLL-7*-2A-*SbCHS-2* was *CaMV 35S* promoter (green) and terminator from pea rbcS-E9 gene (Te9) (highlighted in light green). *SbCHI*-2A-*SbFNSII-2* was under the control of *AtUBQ10* promoter (orange) and terminator of nopaline synthase gene (Tnos) (highlighted in light blue).

Table S1. Primers used for multigene assembly

Primers for Round I of multigene assembly

Primer	Sequences (5' to 3')
**PBI121-PD7-F**	tatgaccatgattacgccaagcttATAGGCAACCGTGGACTTCTTC
**PBI121-PD7-R**	gctcaactccatTTGAGGCTAGGTTTTAGTAGTGAAG
**PD7-SbCYP2D1.1-F**	agcctcaaATGGAGTTGAGCTCTGTCATCTATG
**PD7-SbCYP2D1.1-R**	tcttcatcttcatatTAATATAGAGTAGGTGACAACCTTGG
**SbCYP2D1.1-Thsp-F**	aatcgtttaATATGAAGATGAAGATGAAATATTTG
**SbCYP2D1.1Thsp-R**	aaaacgacggccagtgaattcCTTATCTTTAATCATATTCCATA
**PBI121-SbCLL-7-F**	acgggggactctagaggatccATGGAGAAATCGGGCTATGG
**PBI121-SbCLL-7-R**	tgCTTCAGCAGGCTGAAGTTAGTAGCTCCGCTTCCTAACTTCGATCGGACCTTTTC
**PBI121-SbCHI-F**	aacttcagcctgctgaagCAGGCTGGAGACGTGGAGGAGAACCCTGGACCTATGTCTGCTTCGCCATCCGT
**PBI121-SbCHI-R**	cgatcggggaaattcgagctcTTAAAACAACTCCGATAGTCTTGC
**PBI121-SbCHS-2-F**	acgggggactctagaggatccATGGTGACAGTTGAAGAATTCCAC
**PBI121-SbCHS-2-R**	ggcctgTTTCAAGAGAGAAAAATTAGTGGCACCTGAACCATTGAGAGGCACACTATGC
**PBI121-SbFNSII-2-F**	cttgaaaCAGGCCGGTGACGTTGAAGAGAACCCAGGTCCAATGGAAGTCACACTGAATGTGG
**PBI121-SbFNSII-2-R**	cgatcggggaaattcgagctcTCAGTGCCCGGAAATAACCC

[a] The homology arm sequences are noted in red; All restriction enzyme sites are underlined.

[b] The sequence of P2A polypeptides are noted in green.

Primers for Round II of multigene assembly

Primer	Sequences (5' to 3')
**1300-35S-F1**	ctatgacatgattacgaattcTGAGACTTTTCAACAAAGGGT
**1300-35S-R1**	gcccgatttctccatTGTTCTCTCCAAATGAAATGAACTTC
**35S-CLL-CHI-F1**	tggagagaacaATGGAGAAATCGGGCTATGGC
**35S-CLL-CHI-R1**	gaacgaaagctTTAAAACAACTCCGATAGTCTTGCC
**1300-Te9-F1**	ttgttttaaAGCTTTCGTTCGTATCATCG
**1300-Te9-R1**	acgacggccagtgccaagcttTTGATGCATGTTGTCAATCAAT
**1300-UBQ-F1**	ctatgacatgattacgaattcGTCGACGAGTCAGTAATAAACGGC
**1300-UBQ-R1**	cttcaactgtcaccatCTGTTAATCAGAAAAACTCAGATTAATCG
**35S-CHS-FNS-F1**	ctgattaacagATGGTGACAGTTGAAGAATTCCACC
**35S-CHS-FNS-R1**	tttgaacgatcTCAGTGCCCGGAAATAACCC
**1300-Tnos-F1**	gggcactgaGATCGTTCAAACATTTGGCAATAA
**1300-Tnos-R1**	acgacggccagtgccaagcttGATCTAGTAACATAGATGACAC

[a] The homology arm sequences are noted in red; All restriction enzyme sites are underlined.

Primers for Round III of multigene assembly

Primer	Sequences (5' to 3')
**PD7-SbCYP2D1.1-Thsp-F**	ctatgacatgattacgaattcATAGGCAACCGTGGACTTCTTC
**PD7-SbCYP2D1.1Thsp-R**	cctttgttgaaaagtctcaCTTATCTTTAATCATATTCCATAGTCCATACC
**35S-CLL-CHI-Te9-F2**	ttaaagataaTGAGACTTTTCAACAAAGGGTAATATC
**35S-CLL-CHI-Te9-R2**	acgacggccagtgccaagcttTTGATGCATGTTGTCAATCAAT

[a] The homology arm sequences are noted in red; All restriction enzyme sites are underlined.

Primers for Round IV of multigene assembly

Primer	Sequences (5' to 3')
**FCC-F3**	ctatgacatgattacgaattcATAGGCAACCGTGGACTTCTTC
**FCC-R3**	ttactgactcgtcgacCTTGGACTCCCATGTTGGCA
**CF-F3**	aacatgggagtccaagGTCGACGAGTCAGTAATAAACGGC
**CF-R3**	acgacggccagtgccaagcttGATCTAGTAACATAGATGACAC

[a] The homology arm sequences are noted in red; All restriction enzyme sites are underlined.

Table S2. Primers for PCR detection

Primer	Sequences (5' to 3')
SbCYP82D1.1-F	ATGGAGTTGAGCTCTGTCATCTATG
SbCYP82D1.1-R	TAATATAGAGTAGGTGACAACCTTGG
SbCLL-7-F	ATGGAGAAATCGGGCTATGG
SbCLL-7-R	TTATAACTTCGATCGGACCTTTTC
SbCHI-F	ATGTCTGCTTCGCCATCCGT
SbCHI-R	TTAAAACAACTCCGATAGTCTTGC
SbCHS-2-F	ATGGTGACAGTTGAAGAATTCCAC
SbCHS-2-R	TCAATTGAGAGGCACACTATGC
SbFNSII-2-F	ATGGAAGTCACACTGAATGTGG
SbFNSII-2-R	TCAGTGCCCGGAAATAACCC
Hyg-F	TTGGCGACCTCGTATTGGGA
Hyg-R	CAAGACCTGCCTGAAACCGAA

Table S3. Primers for qRT-PCR analysis

Primer	Sequences (5' to 3')
SbCYP82D1.1-qF	TTGACAAAAAGGTGGAGGTT
SbCYP82D1.1-qR	CAACACTCTCGGTCATATCC
SbCLL-7-qF	ATTGAGGCCACCGTTGTATC
SbCLL-7-qR	CGACTTTTGAAACCGTGGAT
SbCHI-qF	AAGGCAGTAATAGAGAACAAACAG
SbCHI-qR	TTAAAACAACTCCGATAGTCTTG
SbCHS-2-qF	TTCGTGATGGATGAGATGAG
SbCHS-2-qR	CCATTGAGAGGCACACTATG
SbFNSII-2-qF	TCACCTATGGCGTCTCCTTC
SbFNSII-2-qR	CAGCTCCTCAGTGACGTTGA
Leactin-qF	CCAGGTATTGCTGATAGAATGAG
Leactin-qR	GAGCCTCCAATCCAGACAC
